# Saliva is suitable for SARS-CoV-2 antibodies detection after vaccination: A rapid systematic review

**DOI:** 10.3389/fimmu.2022.1006040

**Published:** 2022-09-20

**Authors:** Eliete Neves Silva Guerra, Vitória Tavares de Castro, Juliana Amorim dos Santos, Ana Carolina Acevedo, Hélène Chardin

**Affiliations:** ^1^ Laboratory of Oral Histopathology, Faculty of Health Sciences, University of Brasília, Brasília, DF, Brazil; ^2^ Department of Analytical, Bioanalytical Sciences and Miniaturization, École Supérieure de Physique et de Chimie Industrielles (ESPCI) de la Ville de Paris, Paris, France; ^3^ Faculté de Chirurgie Dentaire, Université Paris Descartes Sorbonne 12 Rue de l’École de Médecine, Paris, France

**Keywords:** SARS-CoV-2, antibodies, saliva, IgG, IgA, COVID-19 vaccines

## Abstract

Since the introduction of efficient vaccines anti-SARS-CoV-2, antibody quantification becomes increasingly useful for immunological monitoring and COVID-19 control. In several situations, saliva samples may be an alternative to the serological test. Thus, this rapid systematic review aimed to evaluate if saliva is suitable for SARS-CoV-2 detection after vaccination. For this purpose, search strategies were applied at EMBASE, PubMed, and Web of Science. Studies were selected by two reviewers in a two-phase process. After selection, 15 studies were eligible and included in data synthesis. In total, salivary samples of approximately 1,080 vaccinated and/or convalescent individuals were analyzed. The applied vaccines were mostly mRNA-based (BioNTech 162b2 mRNA/Pfizer and Spikevax mRNA-1273/Moderna), but recombinant viral-vectored vaccines (Ad26. COV2. S Janssen - Johnson & Johnson and Vaxzevria/Oxford AstraZeneca) were also included. Different techniques were applied for saliva evaluation, such as ELISA assay, Multiplex immunoassay, flow cytometry, neutralizing and electrochemical assays. Although antibody titers are lower in saliva than in serum, the results showed that saliva is suitable for antibody detection. The mean of reported correlations for titers in saliva and serum/plasma were moderate for IgG (0.55, 95% CI 0.38-9.73), and weak for IgA (0.28, 95% CI 0.12-0.44). Additionally, six out of nine studies reported numerical titers for immunoglobulins detection, from which the level in saliva reached their reference value in four (66%). IgG but not IgA are frequently presented in saliva from vaccinated anti-COVID-19. Four studies reported lower IgA salivary titers in vaccinated compared to previously infected individuals, otherwise, two reported higher titers of IgA in vaccinated. Concerning IgG, two studies reported high antibody titers in the saliva of vaccinated individuals compared to those previously infected and one presented similar results for vaccinated and infected. The detection of antibodies anti-SARS-CoV-2 in the saliva is available, which suggests this type of sample is a suitable alternative for monitoring the population. Thus, the results also pointed out the possible lack of mucosal immunity induction after anti-SARS-CoV-2 vaccination. It highlights the importance of new vaccination strategies also focused on mucosal alternatives directly on primary routes of SARS-CoV-2 entrance.

**Systematic Review Registration:**

https://www.crd.york.ac.uk/prospero/display_record.php?ID=CRD42022336968, identifier CRD42022336968.

## Introduction

Since the COVID-19 outbreak, the development of vaccines is the highest priority due to the rapid transmission and lethality of SARS-CoV-2. Although the development of a safe and effective vaccine is a long and complicated process that typically takes 10 to 15 years (1), the scientific community turned it into an active and powerful field to develop emerging vaccines at an unprecedented speed (2, 3). The main COVID-19 vaccine type currently available are messenger RNA (mRNA) based, including BioNTech 162b2 mRNA/Pfizer (BNT) and Spikevax mRNA-1273/Moderna (MOD). Soon there were also recombinant viral-vectored vaccines, as Ad26. COV2. S Janssen - Johnson & Johnson (JJ), Vaxzevria/Oxford AstraZeneca (AZD), and inactivated virus approaches, like CoronaVac (Sinovac/Butantan) and Covaxin (Bharat Biotech) (4).

From the urgent introduction of these vaccines anti-SARS-CoV-2 worldwide until now, more than 60% of the world population had already received a full initial protocol of vaccination (5). The public health effect was mostly in the reduction of symptomatic and severe cases, impacting also on proportionate mortality caused by COVID-19 (4, 6–8). In this regard, vaccines anti-COVID-19 proved to be effective in inducing humoral immunity (9). However, antibody titers declined over time after vaccination, with a subsequent reduction in neutralizing activity (10–13).

In this sense, frequent population follow-up on antibody quantification becomes increasingly useful for immunological monitoring and COVID-19 control after vaccination. Serological testing for SARS-CoV-2 antibody is the standard reference, being important to assess immunological responses after both vaccine and infection (14). Nevertheless, the invasive process needed for blood collection can limit its employment as a frequent method. As an alternative, saliva has been reported to be a rich biofluid in the assessment of immunity for several diseases, especially those in which the mouth is a route of infection (8, 15–17). Oral fluid, more commonly named “saliva”, is a complex mixture of salivary gland secretions, gingival crevicular exudate, oral microorganisms, and food debris. Thus, oral fluid is a potential source of immunoglobulins, such as immunoglobulin G (IgG) issued from the blood and reaching the oral cavity by the gingival crevicular fluid and immunoglobulin A (IgA) issued from the salivary glands. The production of secretory IgA reflects mucosal immunity, which may impact COVID-19 transmission in addition to the current reduction of symptomatic and severe cases (18–20). Moreover, saliva collection is easy, non-invasive, and requires relatively simple instructions, representing several advantages over blood samples (19, 21).

Thus, the characterization of coronavirus saliva-specific signatures could provide valuable information towards antibodies anti-SARS-CoV-2 in vaccinated individuals. So, this rapid systematic review aims to verify whether saliva is suitable for SARS-CoV-2 antibody (immunoglobulins) detection after vaccination.

## Methods

A rapid systematic review was undertaken to evaluate whether saliva is suitable for SARS-CoV-2 antibodies detection after vaccination. Rapid systematic reviews are a knowledge generation strategy that provides high evidence in a short timeframe to support clinical and policy decision-making, especially during disease outbreaks (22, 23). Thus, the methodology was systematized as suggested by the PRISMA guideline (24) with some adaptations, such as a shorter search strategy, faster data extraction, and mostly qualitative synthesis (PROSPERO Protocol - CRD42022336968). The purpose is to provide urgent information with reference to the potential of saliva in assessing immunological response after vaccination anti-SARS-CoV-2. In addition, our evidence also contributes to the new discussion on the induction of mucosal immunity.

### Search strategy and inclusion criteria

Electronic search strategies were developed and applied to Embase, PubMed, and Web of Science (Appendix 1). The search included all articles published until May 8, 2022, without language restrictions. The inclusion criteria were based on the PICOS strategy, in which Population (P): Human vaccinated for SARS-CoV-2, infected or uninfected; Intervention (I): Anti-SARS-CoV-2 vaccine; Comparator/control (C): Humans not vaccinated for COVID-19; Outcomes (O): detection of SARS-CoV-2 antibodies in saliva, type of vaccine, type of antibodies, methods and techniques for detection; and Studies (S): observational studies.

### Study selection, data collection and synthesis

The selection was completed in a systematic two-phase process by VTC and JAS. A third author (ENSG) was involved when required to make a final decision. Final selection was always based on the full text of the publication. VTC and JAS collected the required information from each selected article and ENSG cross-checked all data to confirm its accuracy. Primary outcome was the detection of antibodies in saliva, considering correlation analysis of saliva and serum/plasma, and detectable capacity using titers reference values and proportions. Secondary outcomes included comparison of titers levels among variable characteristics. The qualitative synthesis was conducted by grouping and comparing data reported in included studies in relation to primary and secondary outcomes. Additionally, graphics were conducted to better illustrate the outcomes analyzed in each study. For correlation data, the coefficient of each study reporting this analysis were collected and grouped. Then, the mean with standard deviation were calculated without comparison or statistical tests. The GraphPad Prism software version 9.4.0 (GraphPad Software, La Jolla California USA) was used to construct the graphics.

## Results

### Selection and characteristics of studies

**Figure 1:**
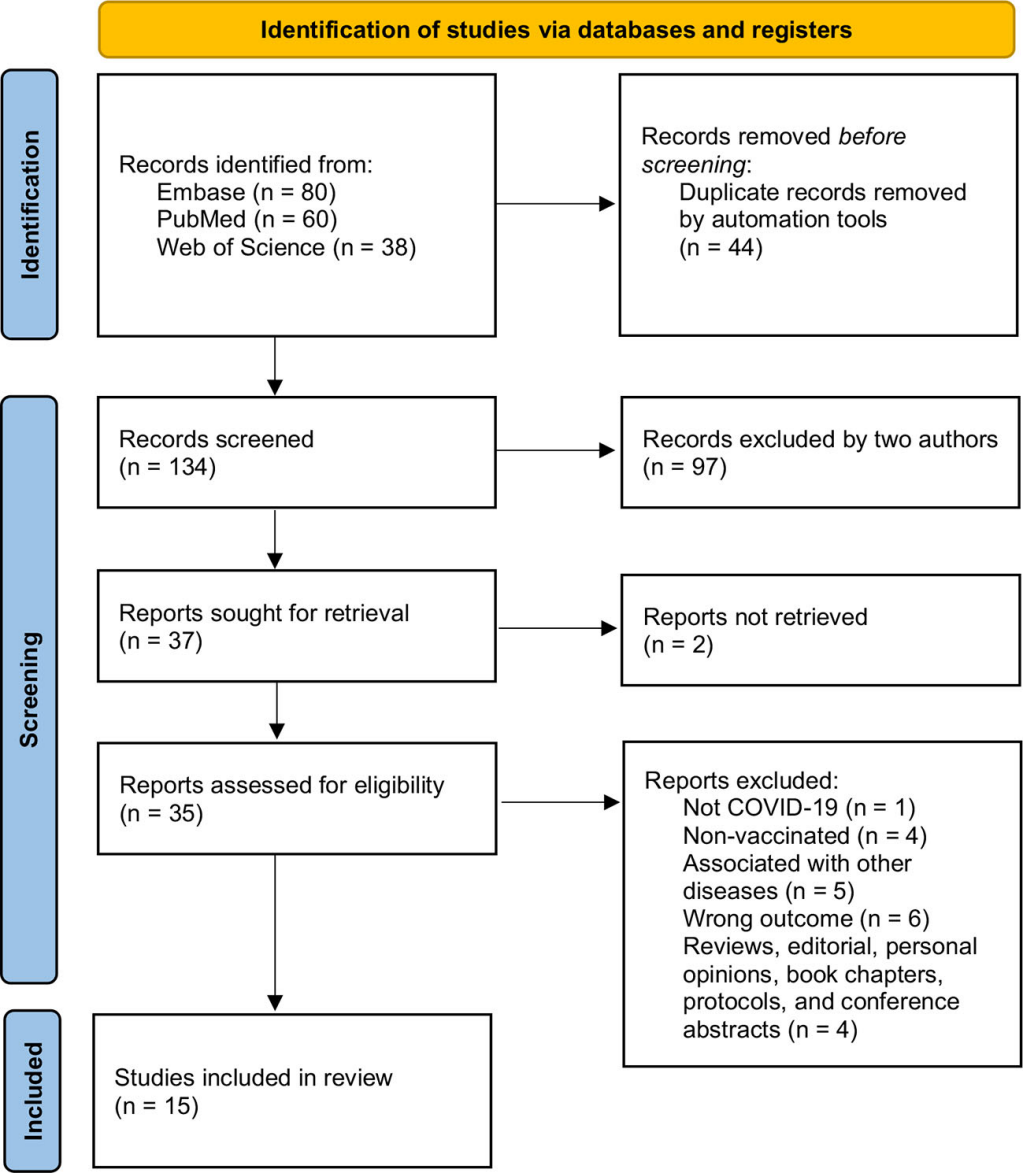
Flow diagram of literature search and selection criteria based on PRISMA 2020.

In the first phase, 178 studies were identified through databases, and after removing duplicates, 134 references remained for screening titles and abstracts. From that, 97 records were excluded and 37 were selected to phase 2. A full-text reading was conducted on 35 references since two were not available. Based on inclusion criteria, 20 articles were excluded, and 15 studies were selected for the synthesis of results (12, 16, 25–37). The flow diagram summarizing the selection process is presented in **Figure 1**.

Of the included articles, 11 are longitudinal studies (12, 16, 25–28, 30, 32, 35–37), and four are cross-sectional studies (29, 31, 33, 34). All studies were published in English between 2021 and 2022. Five of them were conducted in Italy (25, 27, 28, 31, 32), three in United States (12, 16, 33), three in Germany (30, 34, 35), two in Canada (26, 36), one in Croatia (29), and one in Australia (37).

### Summary of results

Considering the 15 included studies, approximately a total of 1,080 vaccinated and/or convalescent individuals were analyzed. The most evaluated vaccine was the BioNTech 162b2 mRNA/Pfizer (BNT) (15 studies, around 637 vaccinated), followed by Spikevax mRNA-1273/Moderna (MOD) (five studies, about 77 vaccinated), Vaxzevria/Oxford AstraZeneca (AZD) (three studies, approximately 44 vaccinated), and Ad26. COV2. S Janssen (JJ) (one study, one vaccinated). Although the sample of main interest consisted of serum/plasma and saliva of vaccinated individuals, some studies also included healthy and previous infected ones as a comparison. From this, only two studies did not include participants previously infected with SARS-CoV-2 (25, 29).

**Figure 2:**
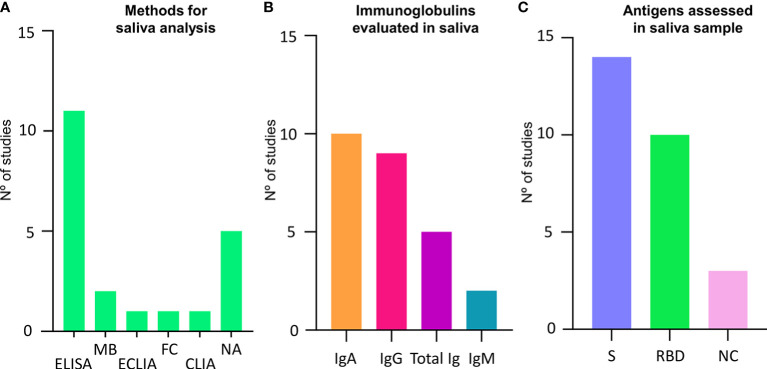
Characteristics of studies and antigen analysis in salivary samples. **(A)** Graphic showing the methods assessed and assays applied for saliva analysis and the number of studies using each type. **(B)** Graphic showing different immunoglobulins assessed and the number of studies assessing each type. **(C)** Graphic showing different antigen assessed for binding reaction and the number of studies assessing each type. CLIA, chemiluminescence immunoassay; ECLIA, electro-chemiluminescence immunoassay; ELISA, Enzyme-linked immunosorbent assay; FC, Flow Cytometry; IgA, Immunoglobulin A; IgG, immunoglobulin G; IgM,Immunoglobulin M; MB, Multiplex Bead; NA, Neutralizing activity; NC, nucleocapsid; NR, Not reported; BD, receptor binding domain; S, spike protein; Total Ig, Total immunoglobulins.

Saliva samples were mainly collected using cotton devices (n=6), such as Salivette^®^ and similar (26, 29, 32, 35–37), splitting methods (n=4) (12, 25, 30, 31) and aspiration (n=1) (27). Some papers did not report the collection method (n=4) (16, 28, 33, 34). The methods of analysis included Enzyme-linked immunosorbent assay (ELISA), Multiplex bead assays, Electro-Chemiluminescence immunoassay (ECLIA), Flow Cytometry (FC), and Chemiluminescence Immunoassay (CLIA). Additionally, five studies evaluated neutralizing activity anti-SARS-CoV-2 (**Figure 2A**).

Saliva samples were evaluated for IgA in 10 studies and for IgG in nine. Total Igs were assessed in five, while IgM was assessed only in two studies (**Figure 2B**). Concerning specific antigens for antibodies detection, 14 studies assessed the spike protein (S) subunits 1 and 2 or whole trimer, 10 studies used the RBD region, and three the nucleocapsid (NC) (**Figure 2C**). Detailed information can be found in **Table 1**.

**Table 1:** Summary of overall descriptive characteristics of included studies (n = 15).

Author/Year/Country	Groups (n)	Age (years)	Antigen tested/antibodies detected (Fluid of collection)	Method analyses (Fluid sublimed to analysis)	Main results
Azzi et al., 2022 (25), Italy	Vaccinated BNT (60)	41.2 ± 10.4 (26-62)	Anti-S/IgG, IgA (Serum and saliva)	ELISA (Serum and saliva)	Pearson correlation of IgG in serum and saliva (r=0.392)
			Anti-S1/S2 IgG (Serum)	CLIA (Serum)	Pearson correlation of IgA in serum and saliva (r=0.291)
				Anti-RBD neutralizing assay (Serum and saliva)	Sensitivity: 99% Specificity: 88%
Darwich et al., 2022 (28), Italy	Vaccinated BNT (92)	38.35 (11.95)	Anti-S Total Ig (Saliva)	ELISA (Saliva)	Spearman correlation of anti-S IgG in serum and saliva (r=0.4)
	Control (19)	42.8 (15.4)	Anti-RBD/S/N IgG, IgA, IgA1, IgA2 (Plasma and saliva)	Adapted dual-ELISA (Plasma and saliva)	Sensitivity 100% Specificity 86.5%
	Unvaccinated and previous infected (28)	47.3 (16.6)			
Garziano et al., 2022 (31), Italy	Vaccinated AZD/BNT (40)	34.1 ± 11.5	Anti-RBD Total Ig (saliva)	ELISA (saliva)	Neutralizing activity titer: All groups
	Vaccinated previous infected (28)	41.36 ± 19.19			Correlation in plasma and saliva (r²=0.32) Vaccinated
	Previous infected (20)	29.4 ± 20.5		Virus neutralization assay (Plasma and saliva)	Correlation in plasma and saliva (r²=0.03) Vaccinated previous infected
					Correlation in plasma and saliva (r²=0.52) Previous infected
					Correlation in plasma and saliva (r²=0.55)
Guerrieri et al., 2021 (32), Italy	Vaccinated BNT (28)	52	Anti-S IgA (Serum and saliva)	ELISA (Serum and saliva)	Production of salivary anti-S1 IgA and anti-RBD IgG
	Previous infected (18)	49 (22-70)	Anti–RBD IgG (Serum and saliva)	CLIA (Serum and saliva)	IgG and IgA production are higher after the vaccine second dose compared to subjects recovered from COVID-19
	Control (33)	52			
Johnson et al., 2022 (12), United States	Vaccinated MOD/BNT/JJ and/or infected (13)	NR	Anti-S IgG (Dried blood and saliva)	ELISA (Dried blood and saliva)	Repeated measures correlation of matrices in blood and saliva (r=0.80)
Ketas et al., 2021 (16), United States	Vaccinated BNT/MOD (85)	39.5	Anti-S/RBD IgM, IgG, IgA (Serum and saliva)	ELISA (Serum and saliva)	Anti-RBD IgA reactivities were higher in saliva than in serum.
	Previous infected (10)				Anti-S IgG were detected in more participants with 2 doses than 1.
	Uninfected (7)				Anti-S-IgA were present in 60.6% saliva samples after 2 doses
Klingler et al., 2021 (33), United States	Vaccinated BNT/MOD (20)	30-69	Anti-S/S1/S2/RBD Total Ig (Plasma and saliva)	Multiplex Bead Ab Binding Assay (Plasma and saliva)	Spearman correlation of anti-S and anti-RBD IgG1 in serum and saliva (r=0.63)
	Previous infected (13)	25-79	Anti-S/RBD IgM, IgG1, IgG1, IgG3, IgG4, IgA1, IgA2 (Plasma and saliva)		
	Control (4)	NR			No correlation found between serum and saliva to IgA1 and IgM
Lapic et al., 2021 (29), Croatia	Vaccinated BNT (43)	52 (27-63)	Anti-S Total Ig (Serum and saliva)	ECLIA (Serum and saliva)	Spearman correlation of Total Ig in serum and saliva (r=0.606)
Meyer-Ardnt et al., 2022 (34), Germany	Elderly vaccinated BNT (18)	83 ± 6	Anti-S1 IgA (Serum and saliva)	ELISA (Serum and saliva)	Spearman correlation of salivary sIgA and neutralization
	Middle-age vaccinated BNT (14)	47 ± 10	Anti-S1 IgG (Serum)	Anti-S1 neutralizing assay (Serum and saliva)	Elderly Vaccinated (r=0.46) in 28d, (r=0.44) in 49d
	Previous infected (37)	36 ± 11			Middle-age vaccinated (r=0.38) in 28d, (r=0.08) in 49d
					Previous infected (r=0.66) in 28d, (r=0.59) in 49d
Nickel et al., 2022 (35), Germany	Vaccinated BNT (104)	41	Anti-S1/RBD IgA (saliva)	Flow cytometry and neutralizing assay by flow cytometry (saliva)	No increase of IgA production at the day of second dose (median 21d) or 14-28 days after second dose was observed in the vaccinated individuals. In contrast, most COVID-19 patients had detectable salivary IgA towards after 15-30 days after the onset of symptoms
	AZD/BNT (11)	31	Anti-S1/NC/IWV IgA, IgG (serum)	ELISA (serum)	
	Previous infected (57)	51	Anti-RBD Polyvalent IgGAM (serum)	ELISpot (serum)	
Pinilla et al., 2021 (30), Germany	Vaccinated 1 dose BNT (22) MOD (7) AZD (13)	NR	Anti-RBD IgG (Serum and saliva)	ELISA (Serum and saliva)	Pearson correlation of IgG in plasma and saliva (r=0.73)
	Previous infected (72)	29 (19–75)		Anti-S1 neutralizing assay (Serum)	
Robinson et al., 2022 (36), Canada	Vaccinated BNT (10)	NR	Anti-S/NC Total Ig (Serum and saliva)	NR	Linearity of anti-S Total Ig in serum and saliva was insignificant 6 months after second dose (p=0.9)
	Previous infected (10)				Sensitivity in 6 months after second dose: 75%
Selva et al., 2021 (37), Australia	Vaccinated BNT (15)	34 (25-57)	Anti-WST/S1/S2/RBD/NC IgA1, IgA2, IgG (Plasma and saliva)	Multiplex bead array (Plasma and saliva)	RBD-specific antibodies were detected in convalescent plasma, however RBD-specific antibodies were not detectable in convalescent saliva in comparison with healthy controls
	Previous infected (16)	52 (22-76)		Anti-S1 neutralizing assay (Plasma and saliva)	Vaccination induced high levels of spike-specific IgG antibodies in tears, saliva and plasma, however no IgA1 and IgA2 responses were detected in saliva
Sheikh-Mohamed et al., 2022 (26), Canada	Vaccinated BNT (66) MOD (34)	48 53	Anti-S/RBD IgA, IgG (Serum and saliva)	ELISA (Serum and saliva)	Spearman correlation of anti-S IgG in plasma and saliva 2-4 weeks after second dose (r=0.63)
	Previous infected (11)			Anti-RBD neutralizing assay by flow cytometry (Serum and saliva)	Spearman correlation of anti-RBD IgG in plasma and saliva 2-4 weeks after second dose (r=0.31)
					Spearman correlation of anti-S IgA in plasma and saliva 2-4 weeks after second dose (r=0.35)
					Spearman correlation of anti-RBD IgA in plasma and saliva 2-4 weeks after second dose (r=0.22)
					Spearman correlation of anti-S IgA and secretory component in the saliva 2-4 weeks after second dose (r=0.42)
					Spearman correlation of anti-RBD IgA and secretory component in the saliva 2-4 weeks after second dose (r=0.45)
					Spearman correlation of anti-S IgA and secretory component in the saliva 6 months after second dose (r=0.53)
					Spearman correlation of anti-RBD IgA and secretory component in the saliva 6 months after second dose (r=0.85)
Terreri et al., 2022 (27), Italy	Vaccinated BNT (34)	46.3 (12.15)	Anti-S IgA (Saliva)	ELISA (Saliva)	S-specific salivary IgA was very low in the majority of vaccinated. Anti-S IgA was still present in the saliva of individuals who had previous COVID-19 infection
	Previous infected (33)	39.9 (11.3)	Anti-NC IgA, IgG, IgM and anti-RBD Total Ig (Serum)	ECLIA (Serum)	
	Control (34)	46.3 (12.15)	Anti-TS IgG (serum)	CLIA (serum)	
				Neutralizing assay by MNA (serum)	

Ab, Antibody; NAb, Neutralizing Antibody; AZD, Vaxzevria/Oxford AstraZeneca; BNT, BioNTech 162b2 mRNA/Pfizer; CLIA, chemiluminescence immunoassay; COVID-19, corona virus diseases 2019; ECLIA, electro-chemiluminescence immunoassay; ELISA, Enzyme-linked immunosorbent assay; ELISpot - Enzyme-linked immune absorbent spot; IgA, Immunoglobulin A; IgG, immunoglobulin G; Total Ig, Total Immunoglobulins; IWV, Inactivated Whole Virion; MNA, micro-neutralization assay; JJ - Ad26. COV2. S, Janssen, Johnson & Johnson; MOD - Spikevax mRNA-1273/Moderna; NC, nucleocapsid; NR, Not reported; r, Correlation coefficient; RBD, Receptor binding domain; S, Spike protein; S1, Spike 1; S2, Spike 2; SARS-CoV-2, severe acute respiratory syndrome corona virus 2; TS, Trimeric spike; WST, Whole spike timer.

### Correlation of immunoglobulins detection in serum/plasma and saliva

**Figure 3:**
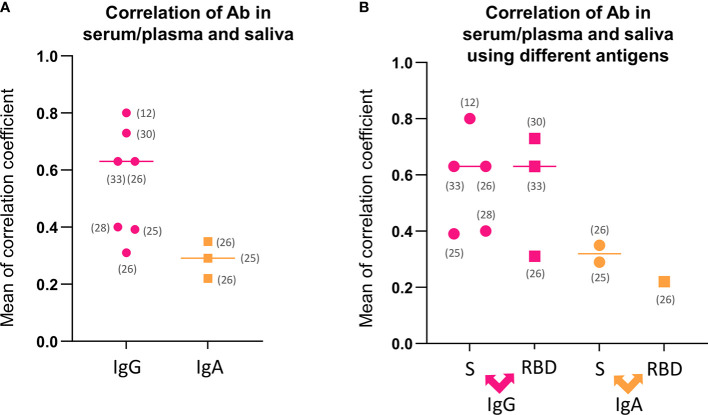
Correlations analysis regarding antibodies detection in serum/plasma and saliva. **(A)** Mean of correlation coefficients between antibodies detection in serum/plasma and saliva for IgG and IgA. **(B)** Mean of correlation coefficients between antibodies detection separated by antigen-antibody reaction for IgG and IgA. IgA, Immunoglobulin A; IgG, immunoglobulin G; RBD, receptor binding domain; S, spike protein; Total Ig, Total immunoglobulins.

To assess the correlation between antibodies quantity in serum/plasma and saliva, seven analyses were reported for IgG, three for IgA, and two for Total Igs. All studies reported linear correlation; however, the results were heterogeneous. The mean coefficients reported for IgG were 0.55 (95% CI 0.38-9.73), indicating a moderated correlation that varies between weak and strong (**Figure 3A**). The mean correlation coefficient for IgA was 0.28 (95% CI 0.12-0.44), representing a weak correlation result (**Figure 3A**). A mean analysis for Total Igs correlation in serum/plasma and saliva was not feasible. The individual reports for Total Igs presented a strong correlation in Lapic et al. (29) study (Spearman correlation; r=0.66), and no correlation in Robinson et al. (36) study (Linearity analysis; p=0.90).

These results lead to a question of whether there is a similarity in correlation strongness when the different antigen-antibody reaction was detected. Thus, the correlations performed with anti-S and anti-RBD were separated, and the mean coefficients were compared for IgG and IgA. The mean analysis showed 0.57 (95% CI 0.35-0.78) for anti-S IgG, 0.55 (95% CI 0.01-1.02) anti-RBD IgG, 0.32 (95% CI -0.06-0.70) for anti-S IgA, and 0.22 for anti-RBD IgA (**Figure 3B**), which suggests approximate results using different antigen binding.

Only three studies reported accuracy values for salivary analysis (**Table 1**). Sensitivity was higher than specificity in two studies (99% and 88%; 100% and 86.5%, respectively). The other study reported just the sensitivity value of 75% six months after the second dose.

### Immunoglobulins titers in saliva

Immunoglobulins levels in serum/plasma and saliva were assessed as an outcome in all included studies. **Table 2** presents the summary of the nine studies that reported antibody titers or proportions detected in saliva versus serum/plasma. There were two ways of reporting: 1. Quantification of antibody titers, and 2. Proportion of individuals with positive detection. The studies presented a main increasing pattern of titers before vaccination, and after first and second doses. On the contrary, Sheikh-Mohamed et al. (26) reported higher detection after one dose than after two doses. Most studies reported higher antibody titers in serum/plasma than in saliva. Six out of nine studies reported the numerical quantification of antibody titers, from which titers’ level in saliva was able to reach the reference value for detection in four studies. The two reminding studies did not define an objective reference value for saliva standardized analysis. Three studies presented proportion values showing that saliva was suitable for antibody detection in all (100%) with superior percentages for IgG than IgA in two studies that assessed both immunoglobins.

**Table 2:** Antibodies titers or proportion of detection in saliva versus serum/plasma of studies that expressly reported numerical data.

Author/Year/Country	Vaccinated group	Before vaccineor infection	1^st^ dose (Period between doseand collection)	2^nd^ dose (Period between doseand collection)	Reference
Azzi et al., 2022 (25), Italy	Saliva	IgG: 0.02 ng/mL IgA: 0.02 ng/mL	(2w) IgG: 0.07 ng/mL IgA: 0.05 ng/mL	(2w) IgG: 10.8 ng/mL IgA: 0.07 g/mL	IgG: 1.54 ng/mL
	Serum	IgG: 0.04 ng/mL IgA: 0.02 ng/mL	(2w) IgG: 432.1 ng/mL IgA: 1.71 ng/mL	(2w) IgG: 20373.65 ng/mL IgA: 49.59 ng/mL	IgG: 904.5 ng/mL
Garziano et al., 2022 (31), Italy	Saliva (ELISA)	NR	NR	(0.5-12m) Vaccinated Total Ig: 30.58%*	Negative < 20%
				(0.5-12m) Vaccinated and previous infected Total Ig: 58.40%*	
	Serum	NR	NR	NR	
Guerrieri et al., 2021 (32), Italy	Saliva	IgG-RBD 1.19 BAU/mL	IgG-RBD (<70d) Previous infected 1 BAU/mL	IgG-RBD (15d) 1.57 BAU/mL	CLIA > 1.19 BAU/mL
		IgA-S1 10.50 COI	IgA-S1 (<70d) Previous infected 13.75 COI	IgA-S1 (15d) 44 COI	ELISA Negative < 0.8 COI Positive >10.50 COI
	Serum	IgG-RBD 0.75 BAU/mL	IgG-RBD (70d) Previous infected 109.10 BAU/mL	IgG-RBD (15d) 1711 BAU/mL	CLIA >4.33 BAU/mL
		IgA-S1 29.29 COI	IgA-S1 (<70d) Previous infected 169.70 COI	IgA-S1 (15d) 739.30 COI	ELISA Negative < 0.8 COI Positive >1.1 COI
Johnson et al., 2022 (12), USA	Saliva	NR	NR	(≤6m) Vaccinated IgG: 29 ng/mL - Peak	NR
				(≤6m) Vaccinated Previous Infected IgG: 982.5 ng/mL- Peak	
	Serum	NR	NR	(≤6m) Vaccinated IgG: 60.1 μg/mL- Peak	NR
				(≤6m) Vaccinated Previous Infected IgG: 532.8 μg/mL- Peak	
Ketas et al., 2021 (16), USA	Saliva	NR	Proportions of detectionS-proteinBNT IgA: 17% IgG: 33% IgM: 0%	Proportions of detection S-protein BNT IgA: 55% IgG: 100% IgM: 17%	NR
			MOD IgA: 71% IgG: 86% IgM: 14%	MOD IgA: 85% IgG: 100% IgM: 8%	
				RBD BNT IgA: 83% IgG: 100% IgM: 4%	
				MOD IgA: 77% IgG: 100% IgM: 8%	
	Serum	NR	Proportions of detectionS-proteinBNT IgA: 38% IgG: 54% IgM: 17%	Proportions of detection S-protein BNT IgA: 100% IgG: 100% IgM: 71%	NR
			MOD IgA: 100% IgG: 100% IgM: 71%	MOD IgA: 100% IgG: 100% IgM: 62% RBD	
				BNT IgA: 76% IgG: 100% IgM: 55%	
				MOD IgA: 100% IgG: 100% IgM: 46%	
Lapic et al., 2021 (29), Croatia	Saliva	NR	NR	(71d) Total Ig: 2.5 U/mg proteins	NR
	Serum	NR	NR	(71d) Total Ig: 1274 U/mL	Negative < 0.8 U/mL
Pinilla et al., 2021 (30), Germany	Saliva	NR	(1m) IgG: 626 ng/mL Proportions of detection (1y after infection) IgG: 72% (15m after infection) IgG: 80%	NR	NR
	Serum	NR	(1m) IgG: 1458 µg/mL Proportions of detection (4m after infection) IgG: 89% (12m after infection) IgG: 89% (15m after infection) IgG: 98%	NR	NR
Robinson et al., 2022 (36), Canada	Saliva	Total Ig: <0.4 U/mL	Total Ig <0.4 U/mL	Total Ig (56d) <0.4 U/mL (70d) 14.3 U/mL (86d) 11.2 U/mL (>6m) 2.6U/mL	<0.4 U/mL Negative
	Serum	NR	NR	Total Ig (56d) 79 U/mL (>6m) 1558 U/mL (70d) >2500 U/mL (86d) >2500 U/mL (>6m) 1558 U/mL	NR
Sheikh-Mohamed et al., 2022 (26), Canada	Saliva	NR	Proportion of detection (2w) Anti-S-IgG: 97% (2w) Anti-RBD- IgG: 52%	Proportion of detection (NR) Anti-S-IgG: 94% (NR) Anti-RBD-IgG: 93%	NR
			(2w) Anti-S-IgA: 93% (2w) Anti-RBD-IgA: 41%	(NR) Anti-S-IgA: 41% (NR) Anti-RBD-IgA: 20%	
	Serum	NR	NR	NR	NR

BAU, Binding Antibody Units; BNT, BioNTech 162b2 mRNA/Pfizer; CLIA - Chemiluminescence Immunoassay; COI, Cut off Index; d - Days; ELISA, Enzyme-linked Immunosorbent Assay; IgA, Immunoglobulin A; IgG, Immunoglobulin G; IgM, Immunoglobulin M; mL, milliliter; m - Months; MOD, Spikevax mRNA-1273/Moderna; ng, nanogram; NR, Not Reported; RBD, Receptor Binding Domain; S, Spike protein; U, Units; µg, microgram; WD, Wild type; y - years: *Data calculated by authors based on reported information. In parentheses is the time between collection and the first dose, second dose, or infection, when it is reported in the study.

**Figure 4:**
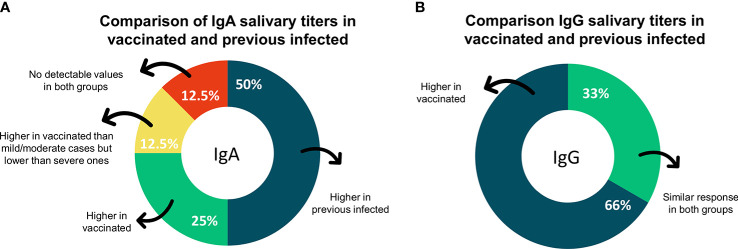
The proportion of studies on secondary outcomes. **(A)** Graphic showing the proportion of studies reporting results on IgA comparison between vaccinated and previous infected individuals **(B)** Graphic showing the proportion of studies reporting results on IgG comparison between vaccinated and previous infected individuals. IgA, immunoglobulin A; IgG, Immunoglobulin G.

Furthermore, eight studies performed the comparison of IgA titers in saliva between vaccinated and previous infected individuals with or without vaccine doses. Four studies reported lower IgA salivary titers in vaccinated without previous infection (50%), otherwise, two reported higher titers of IgA in those individuals (25%%). One reported conflicting results showing higher titers in vaccinated compared to mild/moderate COVID-19 cases and lower titers compared to severe ones (12.5%). In addition, one study failed to detect values for both groups of individuals (12.5%) (**Figure 4A**). Toward IgG, two studies reported high levels in saliva of only vaccinated individuals compared to previous infected ones (66%) and one reported similar results for vaccinated and infected (33%) (**Figure 4B**).

### Neutralizing activity

Two studies reported the main conclusions on neutralization correlations (**Table 1**). Garziano et al. (31) assessed the correlation of neutralizing activity in plasma and saliva. The reported results showed stronger coefficients for individuals who were previously infected with (r=0.52) or without (r=0.55) vaccination, compared to those uninfected and vaccinated (r=0.03). Meyer-Ardnt et al. (34) evaluated the correlation of salivary secretory IgA and neutralizing activity, showing weak results for elderly vaccinated individuals (r=0.46 after 28 days; r=0.44 after 49 days), very weak for middle-aged vaccinated (r=0.38 after 28 days; r=0.08 after 49 days), and moderate for previous infected ones (r=0.66 after 28 days; r=0.59 after 49 days). Additionally in neutralizing fields, Nickel et al. (35) was the only study reporting neutralization after the third dose of vaccine. The results provided evidence of stronger neutralizing activity in the group receiving heterologous vaccination protocol (AZD-BNT) compared to homologous one (BTN-BNT). Thus, a combination of different SARS-CoV-2 vaccine classes seems to lead to a stronger humoral immune response which may result in a better protective effect.

## Discussion

SARS-CoV-2 is an enveloped single-stranded RNA virus, of which surface glycoprotein spike mediates viral entrance into host cells, especially through angiotensin-converting enzyme 2 (ACE2) (38). Against it, antibodies modulate cell infection by neutralizing viral antigen binding (39). Potent neutralizing antibodies were readily isolated from convalescent individuals, suggesting that SARS-CoV-2 is a neutralization-sensitive virus. Those neutralizing antibodies are targeted against the RBD motif of the spike protein, which is a relevant antigen to vaccines goals (40, 41). In this context, vaccine based on mRNA anti-SARS-CoV-2 was the most reported in this review. It demonstrated the capability of inducing antibodies in both previously infected and not infected individuals, increasing humoral and cellular immunity after the second vaccination dose (42). Furthermore, mRNA vaccines encode trimerized RBD which is modified by adding a “foldon” trimerization domain to increase immunogenicity. The result is the induction of anti-RBD neutralizing antibodies specific for SARS-COV-2 in plasma, and a T cell response with Th1 cytokine and low-level CD8 T cell (43).

In this systematic review, the main outcome was the correlation between serum/plasma and saliva antibodies, with the purpose of comparing the two types of samples source. Among the techniques for detecting antibodies, ELISA is one of the most used for serological tests (44). For years, its use has been also reported for saliva samples in different disease responses, such as hepatitis A (45), leprosy (46), and autism spectrum disorder (47). Thus, ELISA is suggested as a method to detect anti-COVID-19 antibodies in saliva. In this view, as most SARS-CoV-2 vaccines in use or in advanced development are based on the viral spike protein subunits, the antigen used for antibody detection is the S protein or its RBD region. Protein S is present on virions as prefusion trimers, where RBD is arbitrarily open or closed (41). IgG, mainly IgG1, dominates S- and RBD-specific antibody responses, which are intended against structurally folded S and RBD and three distinct peptide epitopes in S2. Although immunity assessment assays may vary respecting antigen-antibodies reactions, the synthesis of results suggests that it does not impact antibody detection after vaccination.

From our results, the mean correlation coefficient between serum/plasma and saliva was stronger for IgG than IgA. Nevertheless, at mucosal sites secretory IgA act with an essential role in protection mucosal surfaces by preventing the binding of viruses to epithelial cells (48). Salivary IgG, as well as a very limited amount of monomeric IgA, are derived from plasma *via* gingival crevices and could participate in viral protection (28, 49). Some articles comparing the immune responses of vaccinated and previously infected individuals suggested that salivary IgA titers were higher in the saliva of the infected, whereas IgG presented a higher salivary titer in vaccinated individuals (25, 27, 28, 35). As the current anti-COVID-19 vaccines used a systemic injection, they predominantly induce circulatory IgG (20, 50) indicating that after a mRNA vaccination, the IgG are translocated into saliva in sufficient amounts to have a high predictive value of induced seroconversion. However, as the testing methods used only the S protein (or its RDB subunit) as antigens, it is difficult to compare the IgG titers obtained after vaccination or natural infection. Indeed, except for vaccines that used inactivated viruses, the vaccinal antigen is based on the S protein, and only anti-S antibodies were obtained after vaccination, whereas the natural infection induces various antibodies specific to the various proteins of the virus (39). Thus, the testing methods are built to evidence the performance of the vaccination rather than the complexity of the antibody response obtained after natural infection. For IgA, several reports concluded that SARS-CoV-2 infection was associated with a mucosal secretory IgA response (25, 48) whereas, in vaccinated individuals, the IgA present in saliva were from blood origin (28). Thus, salivary IgAs induced by vaccination seems to be mainly exuded from serum while in previous infected individuals it came from a local mucosal immunity response.

Neutralizing antibodies were also assessed in the included studies. Viral neutralization plays a key role in anti-viral immunity and assessing its capacity is an important strategy to measure protective immunity (43, 51). In serum, neutralizing activity seems to be similar in previously infected individuals, either vaccinated or not, and uninfected vaccinated ones. However, neutralizing activity in saliva was high in convalescents and scarcely detected after vaccination (31). This observation could be explained by the absence of mucosal immunity induction in vaccinated individuals, associated with a quantity of Ig issued from the crevicular fluid that was insufficient to be neutralizing. Furthermore, one dose of vaccine was able to boost an anti-SARS-CoV-2 response in previously infected individuals, whilst the third dose with a different vaccine type led to a significantly stronger response than only two doses (31, 35). Although a neutralizing activity was detected in saliva, intramuscular vaccines are not proved to be effective in producing salivary effects.

Besides the protection against the severe form of COVID-19, it is also essential to understand whether and how vaccination can decrease SARS-CoV-2 transmission (52). According to included studies, available data provided a weak response of intramuscular vaccines to elicit readily detectable mucosal immunity, suggesting the importance of local induction. Considering the respiratory tropism of the SARS-CoV-2 virus, a vaccine delivered intranasally would be useful to induce mucosal immunity directly at the port of virus entrance, also preventing transmission (53). Furthermore, nasal immunization is better than parenteral routes when seeking to achieve mucosal immunity, since the capability to induce IgA specific for SARS-CoV-2 in the respiratory tract may avoid virus spreading to the lung and avert respiratory problems (54). In this field, orally and intranasally administered vaccines have already been approved for humans against various mucosal pathogens (55). Currently, at least 12 projects are presenting intranasal candidates anti-SARS-CoV-2 at pre-clinical or clinical phases (56). In addition to potentially inducing sterilizing immunity, intranasal alternatives for COVID-19 vaccines are predominantly focused on viral vectors and protein subunits, representing safer delivery platforms than the whole pathogens used in all the licensed mucosal vaccines (55, 56). However, the development of a new safe, and an efficient mucosal vaccine is a complex process and several factors including antigen doses, formulation, administration route and adjuvants should be considered (54, 57). Thus, the kinetics and durability of the mucosal responses are also key factors in vaccine development. As mucosal vaccines seem to be potential alternatives for decreasing SARS-CoV-2 transmission, many efforts may focus on those strategies development turning into a current field of interest.

There are some limitations to be highlighted. First, the used techniques for antibodies detection in saliva were validated for serum analysis, resulting in difficulties to establish reference values for saliva quantification in some studies. Second, the included studies are highly heterogeneous with respect to samples, collection intervals, and strategies for reporting data. Third, in some studies, the sample size was not well defined for all reported results, varying during the collection and analysis period process. Lastly, as COVID-19 is an urgent field, especially on vaccination at this time, we also included pre-print studies and letters for the editor, which impact detailed collection and quality.

## Conclusion

Saliva is a suitable biofluid alternative for anti-SARS-CoV-2 antibodies detection in vaccinated and in previously infected individuals. Although salivary antibody titers are lower than serum titers, the detection of anti-SARS-CoV-2 immunoglobulins in saliva are satisfactory. Concerning specific immunoglobulins in vaccinated individuals, saliva seems to frequently present IgG but not uniformly IgA. The mean correlations in serum/plasma and saliva were moderate for IgG and weak for IgA. Thus, the results also suggest and pointed out the possible lack of mucosal immunity induction after anti-SARS-CoV-2 vaccination. It highlights the importance of new vaccination strategies focused also on mucosal alternatives directly on primary routes of SARS-CoV-2 entrance.

## Data availability statement

The original contributions presented in the study are included in the article/**Supplementary Material**. Further inquiries can be directed to the corresponding author.

## Author contributions

EG, AA, and HC designed the study. EG, VC, and JA wrote the first draft of the manuscript. JA conducted the search strategy. VC and JA selected the articles. EG was involved when required to make a final decision. VC and JA collected the required information from each selected article and EG cross-checked all data to confirm its accuracy. AA and HC reviewed and revised the draft. All authors contributed to the article and approved the submitted version.

## Funding

This study received financial support by COPEI-DPI/DEX, University of Brasilia. It was supported by FAPDF (Foundation for Research Support of the Federal District, Brazil, *Edital* 04/2021 (PI: Guerra - 00193-00001145/2021-12). VC and JA are supported by CAPES (Coordination for the Improvement of Higher Education Personnel), Brazil. EG is supported by the CNPq (National Council for Scientific and Technological Development), Ministry of Education, Brazil.

## Conflict of interest

The authors declare that the research was conducted in the absence of any commercial or financial relationships that could be construed as a potential conflict of interest.

## Publisher’s note

All claims expressed in this article are solely those of the authors and do not necessarily represent those of their affiliated organizations, or those of the publisher, the editors and the reviewers. Any product that may be evaluated in this article, or claim that may be made by its manufacturer, is not guaranteed or endorsed by the publisher.
