# Supplementary material for: Saliva is suitable for SARS-CoV-2 antibodies detection after vaccination: A rapid systematic review
Source: Front Immunol. 2022 Sep 20;13:1006040. doi: 10.3389/fimmu.2022.1006040 (PMC9530471; doi:10.3389/fimmu.2022.1006040)
Supplement: Supplementary file 1 [file Table_1.pdf]

Supplementary Material

Table 1. Search strategies with appropriated key words and MeSH terms.

Database	Search strategies<br>(Search date: May 08, 2022)	References
Embase	('salivary' OR 'saliva'/exp OR 'saliva') AND ('immunoglobulins'/exp OR 'immunoglobulins' OR 'antibody'/exp OR 'antibody' OR 'immunoglobulin'/exp OR 'immunoglobulin' OR 'immune globulins'/exp OR 'immune globulins' OR 'antibodies'/exp OR 'antibodies' OR 'antigen-antibody reactions'/exp OR 'antigen-antibody reactions' OR 'antigen-antibody complex'/exp OR 'antigen-antibody complex' OR 'antibody-producing cells'/exp OR 'antibody-producing cells' OR 'antibody specificity'/exp OR 'antibody specificity' OR 'igm'/exp OR 'igm' OR 'igg'/exp OR 'igg' OR 'iga'/exp OR 'iga') AND ('2019 ncov'/exp OR '2019 ncov' OR 2019ncov OR '2019 novel coronavirus'/exp OR '2019 novel coronavirus' OR 'covid19'/exp OR covid19 OR 'new coronavirus' OR 'novel coronavirus' OR 'sars cov-2'/exp OR 'sars cov-2' OR (('wuhan'/exp OR wuhan) AND ('coronavirus'/exp OR coronavirus)) OR 'covid 19'/exp OR 'covid 19' OR 'sars-cov'/exp OR 'sars-cov' OR '2019-ncov'/exp OR '2019-ncov' OR 'sars-cov-2'/exp OR 'sars-cov-2' OR 'coronavirus' OR 'coronavirus'/exp OR coronavirus OR 'covid'/exp OR covid OR 'covid-19'/exp OR 'covid-19' OR 'sars'/exp OR sars) AND ('vaccines'/exp OR 'vaccines' OR 'vaccine'/exp OR vaccine)	80
PubMed	("salivary" OR "saliva" OR "saliva"[MeSH Terms] OR saliva[Text Word]) AND ("2019 nCoV" OR 2019nCoV OR "2019 novel coronavirus" OR "COVID 19" OR COVID19 OR "new coronavirus" OR "novel coronavirus" OR "SARS CoV-2" OR (Wuhan AND coronavirus) OR "COVID 19" OR "SARS-CoV" OR "2019-nCoV" OR "SARS-CoV-2" OR "coronavirus"[MeSH Terms] OR coronavirus[Text Word] OR coronavirus OR COVID OR "COVID-19" OR SARS[All Fields]) AND ("immunoglobulins"[MeSH Terms] OR "antibodies"[MeSH Terms] OR antibody[Text Word] OR "Immunoglobulin" OR "Immune Globulins" OR "Antibodies" OR "Antigen-Antibody Reactions" OR "Antigen-Antibody Complex" OR "Antibody-Producing Cells" OR "Antibody Specificity" OR "IgM"[All Fields] OR "IgG"[All Fields] OR "IgA"[All Fields]) AND ("vaccines"[MeSH Terms] OR vaccine[Text Word] OR ("sars-cov-2"[MeSH Terms] OR "covid-19"[MeSH Terms] OR covid[Text Word]) AND ("vaccines"[MeSH Terms] OR vaccine[Text Word]))	60
Web of Science	(TS=("salivary" OR "saliva")) AND (TS=("2019 nCoV" OR "2019nCoV" OR "2019 novel coronavirus" OR "COVID 19" OR "COVID19" OR "new coronavirus" OR "novel coronavirus" OR "SARS CoV-2" OR (Wuhan AND coronavirus) OR "SARS-CoV" OR "2019-nCoV" OR "SARS-CoV-2" OR "coronavirus" OR "COVID" OR "COVID-19" OR SARS)) AND (TS=("immunoglobulins" OR "antibody" OR "Immunoglobulin" OR "Immune Globulins" OR "Antibodies" OR "Antigen-Antibody Reactions" OR "Antigen-Antibody Complex" OR "Antibody-Producing Cells" OR "Antibody Specificity" OR "IgM" OR "IgG" OR "IgA")) AND (TS=("vaccines" OR "vaccine"))	38
